# A Rare Presentation of Late-Onset Idiopathic Pulmonary Hemosiderosis: A Case Report

**DOI:** 10.7759/cureus.57001

**Published:** 2024-03-26

**Authors:** Sanjay Gabhale, Arun Balan, Vishnu Prabhakar, Mithun Nilgiri K

**Affiliations:** 1 Respiratory Medicine, Dr. D. Y. Patil Medical College, Hospital and Research Centre, Dr. D. Y. Patil Vidyapeeth, Pune, IND

**Keywords:** diffuse alveolar hemorrhage, hemosiderin-laden macrophages, transbronchial lung biopsy, bronchoalveolar lavage, fiberoptic bronchoscopy, hemoptysis, iron deficiency anemia, idiopathic pulmonary hemosiderosis

## Abstract

Idiopathic pulmonary hemosiderosis (IPH) is a rare cause of diffuse alveolar hemorrhage (DAH). It is associated with a high mortality rate and recurrent episodes of widespread alveolar hemorrhage and most commonly affects children. Here, we present a rare occurrence of late-onset idiopathic pulmonary hemosiderosis in a 74-year-old male. He was admitted for non-resolving pneumonia, hemoptysis, and type 1 respiratory failure, along with sideropenic anemia. Chest imaging showed bilateral upper lobe and right middle lobe alveolar opacities. Infective and autoimmune etiologies of diffuse alveolar hemorrhage were ruled out during the evaluation. Transbronchial lung biopsy showed patchy alveolar hemorrhage and abundant hemosiderin pigment deposition, revealing idiopathic pulmonary hemosiderosis. The patient was successfully treated with oral steroids, followed by complete radiological resolution without clinical relapse at one-year follow-up.

## Introduction

Idiopathic pulmonary hemosiderosis (IPH) represents a rare pulmonary disorder characterized by diffuse alveolar hemorrhage (DAH) of unknown etiology [1]. While predominantly observed in pediatric populations [2], cases of IPH in adults, particularly in the elderly, are exceedingly rare and pose significant diagnostic challenges [3]. This condition manifests with recurrent episodes of intra-alveolar bleeding, leading to symptoms such as cough, dyspnea, and hemoptysis, often culminating in respiratory failure.

The pathogenesis of IPH remains elusive, with proposed mechanisms including genetic predisposition, autoimmune dysfunction, environmental triggers, and metabolic abnormalities. Despite advancements in diagnostic techniques, establishing a definitive diagnosis of IPH can be arduous due to its nonspecific clinical presentation and overlap with other pulmonary conditions, necessitating a comprehensive evaluation encompassing imaging studies, bronchoscopy, and histopathological examination.

In this report, we delve into the clinical presentation, diagnostic strategies, and therapeutic modalities pertinent to late-onset IPH, offering insights into the nuanced management of this rare pulmonary disorder in the elderly population. Through the comprehensive analysis of this case, we aim to enhance understanding, promote early recognition, and optimize outcomes in similar clinical scenarios, thereby contributing to the evolving landscape of pulmonary medicine.

## Case presentation

A 74-year-old male, a retired factory worker and former smoker, presented with complaints of breathlessness, cough, and loss of weight and appetite. The patient was cured of pulmonary tuberculosis (TB) 12 years ago after completing treatment and was managed as a case of obstructive airway disease. Recently, breathlessness progressed to Modified Medical Research Council (MMRC) grade 3 with intermittent productive cough and hemoptysis of approximately 5-10 mL per episode.

On presentation, the patient was conscious, oriented, and afebrile. The patient had pallor and hypoxemia, maintaining oxygen saturation at 78% on room air and 95% on 2 L of oxygen via nasal prongs. Auscultation revealed right-sided crepitation in mammary and infra-axillary areas.

**Figure 1:**
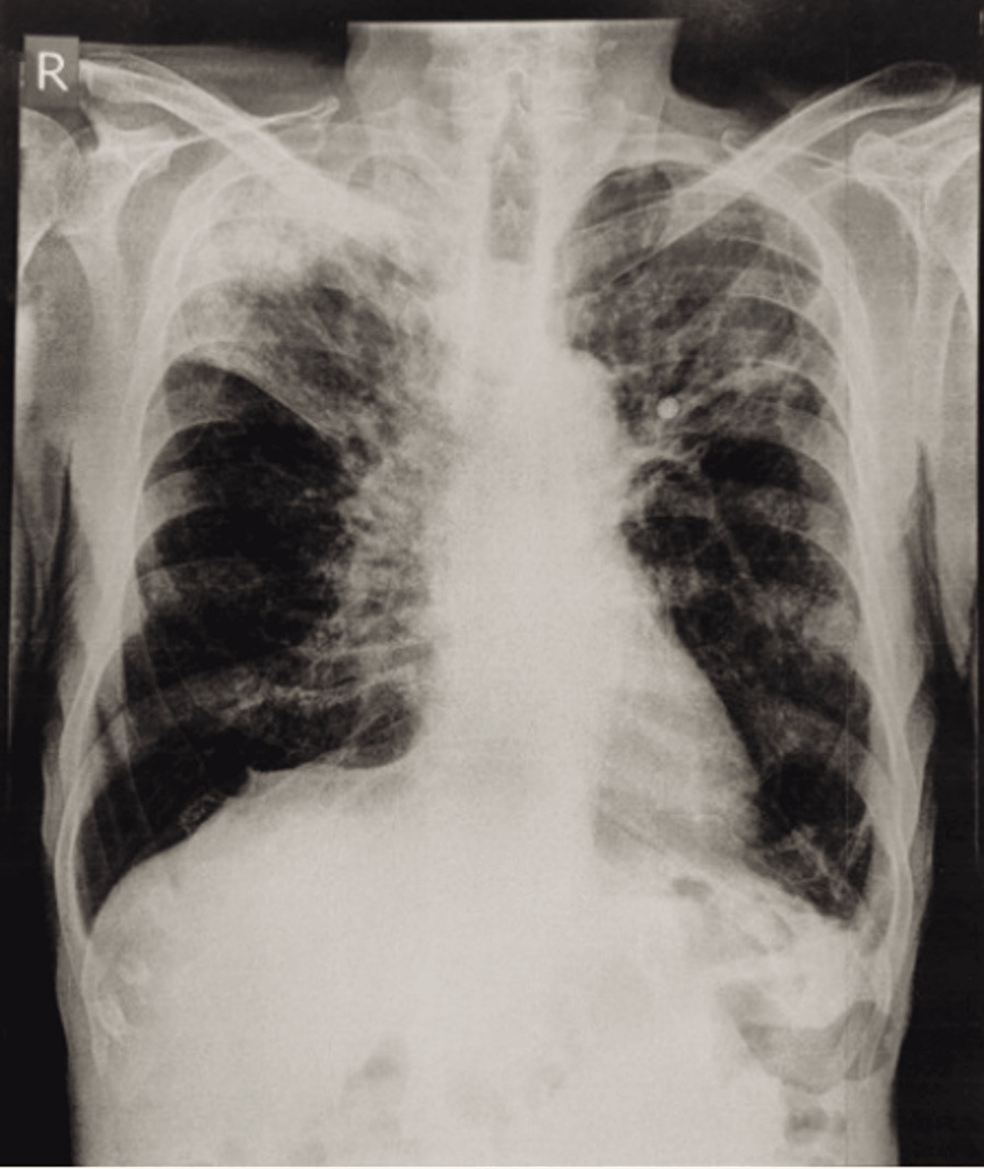
Chest radiograph showed bilateral upper zone and right middle zone inhomogeneous opacities with hyperinflated lung fields

**Figure 2:**
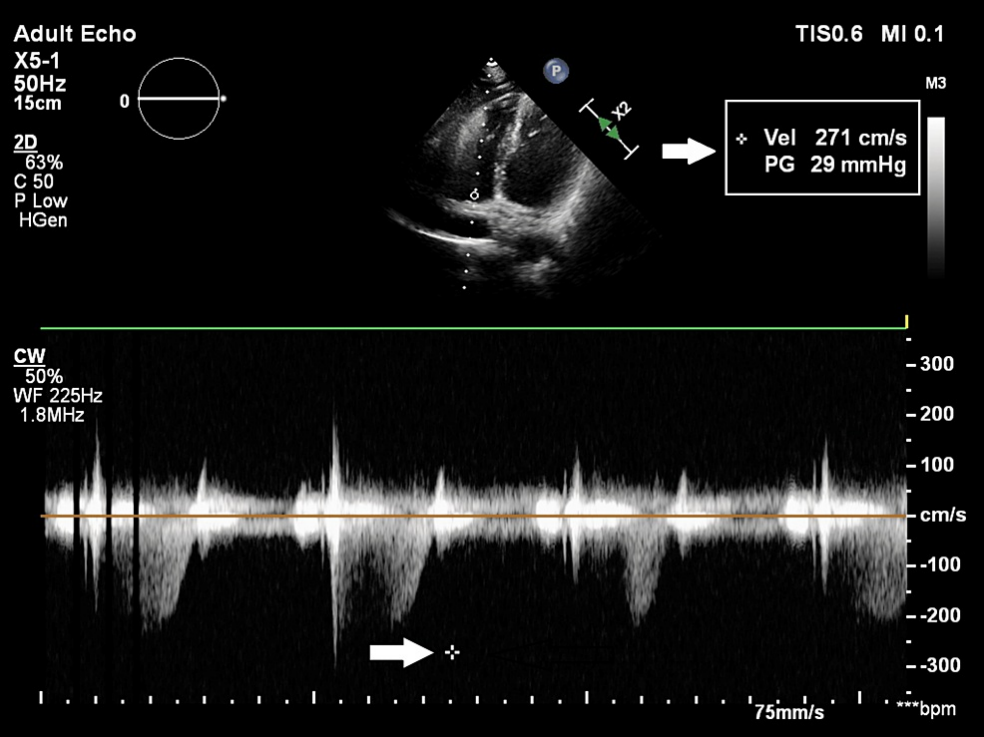
2D echo showing mild pulmonary hypertension The pressure gradient across the tricuspid valve shows 29 mmHg (white arrow). 5 mmHg pressures were added to the tricuspid valve pressure gradient since the inferior vena cava was normal. Right ventricular systolic pressure was derived as 34 mmHg, which indicated mild pulmonary hypertension. 2D echo: two-dimensional echocardiography

Laboratory investigations are mentioned in Table 1. Renal function test (RFT), liver function test (LFT), serum electrolytes, and urine analysis were within normal limits. Among radiological investigations, chest radiographs showed bilateral upper zone and right middle zone inhomogeneous opacities with hyperinflated lung fields (Figure 1). An electrocardiograph (ECG) showed normal sinus rhythm. Two-dimensional echocardiography (2D echo) (Figure 2) showed mild pulmonary hypertension. Arterial blood gas (ABG) analysis was suggestive of type 1 respiratory failure (Table 2).

**Table 1:** Laboratory investigations INR: international normalized ratio

Investigation	Result	Biological reference
Hemoglobin	6.8 g/dL	13.2-16.6 g/dL
Hematocrit	21.5%	38.3%-48.6%
Ferritin	502 ng/mL	21.81-274.66 ng/mL
Serum iron	18 µg/mL	50-150 µg/mL
Transferrin	7.56%	20%-50%
Prothrombin time	12 seconds	10.09-13.79 seconds
INR	1.10	0.85-1.15
D-dimer	2,178 ng/mL	0-500 ng/mL

**Table 2:** ABG suggestive of type 1 respiratory failure ABG: arterial blood gas, pCO2: partial pressure of carbon dioxide, pO2: partial pressure of oxygen

	Observed value	Biological reference interval
pH	7.52	7.35-7.45
pCO2	32 mmHg	35-45 mmHg
pO2	71 mmHg	83-108 mmHg
Bicarbonate	26.1 mEq/L	21-28 mEq/L
Standard bicarbonate	27.6 mEq/L	22-26 mEq/L
Oxygen saturation capacity	96%	95%-98%

**Figure 3:**
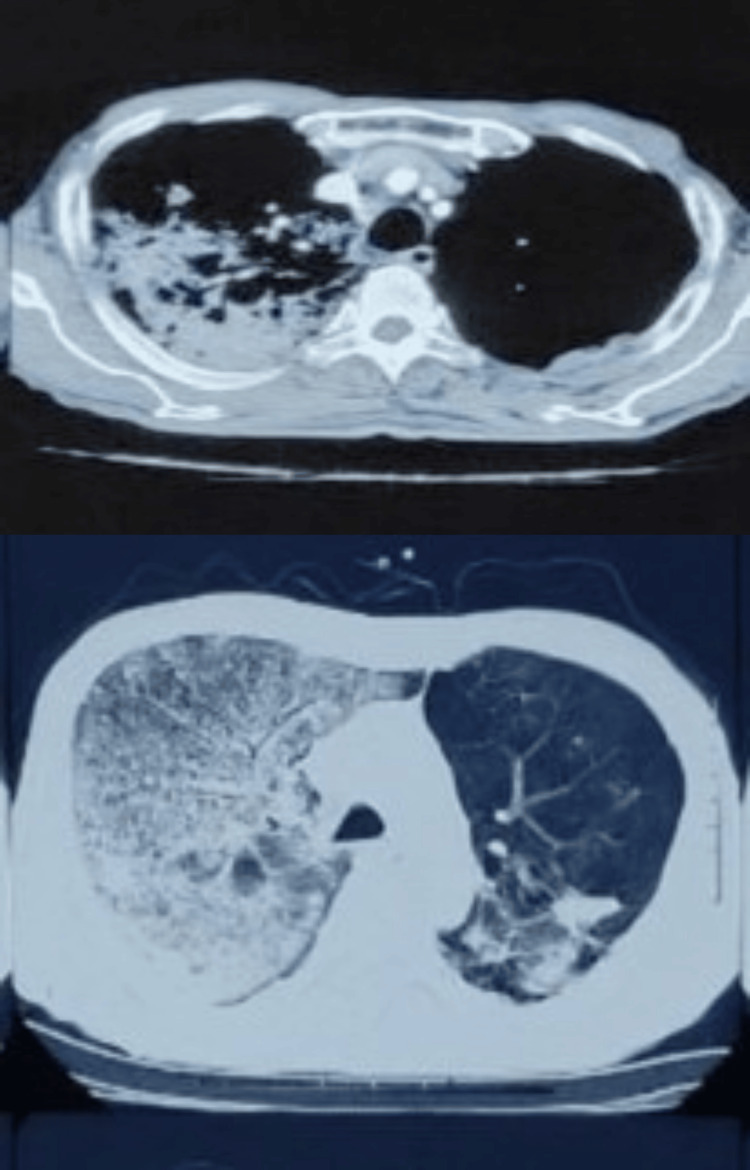
CT showing consolidation and ground glass opacities of the right upper lobe and multiple areas of air trapping seen in the left lung, along with a few fibrotic bands CT: computed tomography

The patient was admitted to the intensive care unit (ICU) and managed with empirical antibiotics, noninvasive ventilation, and supportive treatment. He was given a packed red cell transfusion to correct anemia and shifted to the ward after stabilization. On further evaluation, contrast-enhanced computed tomography (CECT) of the thorax revealed patchy consolidation in the right upper, middle, and left upper lobes. Patchy areas of random ground glass opacities and interstitial septal thickening were seen in the right lower lobe and left upper and lower lobes. Multiple areas of air trapping were seen in the left lung, along with a few fibrotic bands (Figure 3).

**Figure 4:**
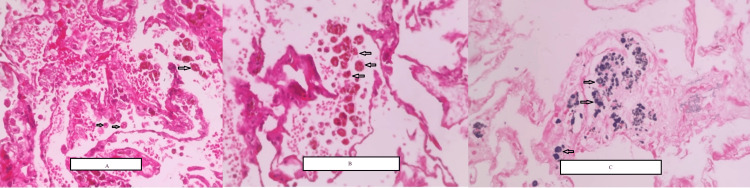
Histopathological examination of the TBLB sample A: Intra-alveolar hemorrhage with hemosiderin-laden macrophages (arrows). B: Hemosiderin-laden macrophages in the alveoli (arrows). C: Prussian blue stain for iron-positive blue deposits in the alveoli (arrows). TBLB: transbronchial lung biopsy

Sputum studies were inconclusive, and hence, fiberoptic bronchoscopy with bronchoalveolar lavage (BAL) combined with transbronchial lung biopsy (TBLB) was done due to the high suspicion of tuberculosis. Sputum Gram stain, Ziehl-Neelsen stain, and GeneXpert came negative, ruling out tuberculosis. TBLB histopathological report showed patchy alveolar hemorrhage with abundant hemosiderin pigment and mild focal interstitial fibrosis (Figure 4). Granulomas, vasculitis, and thromboembolic or fungal elements were not seen.

**Figure 5:**
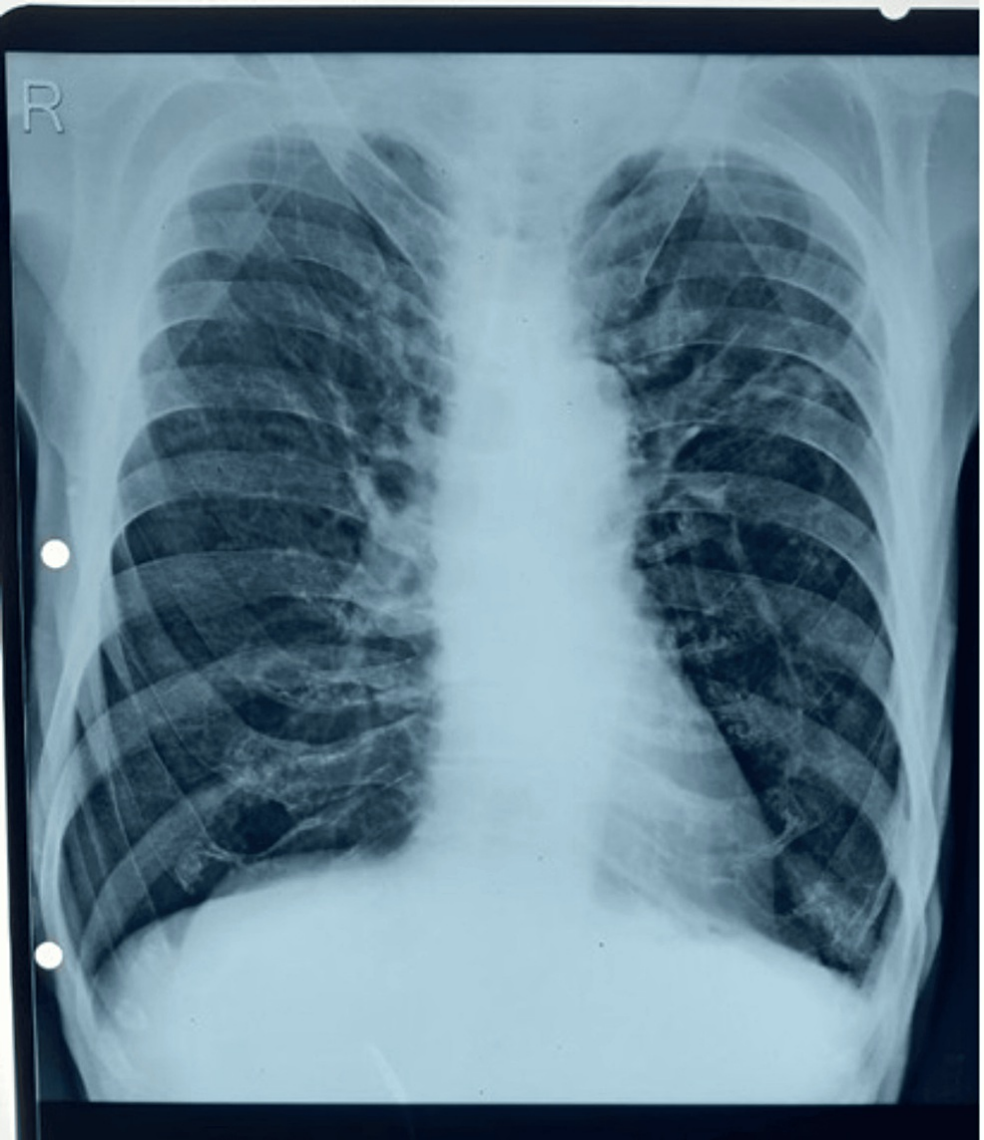
Chest radiograph showing complete radiological resolution after starting steroids

The patient tested negative for antineutrophil cytoplasmic antibodies (C-ANCA) and antinuclear antibody (ANA) detection by indirect immunofluorescence technique, ruling out vasculitis and other connective tissue disorders. He was started on tablet methylprednisolone 16 mg twice daily for one month and tapered over the next two months, to which he responded with symptomatic improvement. He was discharged from the hospital on day 20 and followed up periodically over a period of one year. He remained asymptomatic during follow-up visits with complete radiological resolution after five months (Figure 5).

## Discussion

Idiopathic pulmonary hemosiderosis (IPH) is a rare disorder, and its prevalence and incidence in the general population are unknown. Many previously diagnosed IPH cases may have been misclassified as diffuse alveolar hemorrhage (DAH) due to limited diagnostic scope as noted by Ioachimescu et al. [1]. About 80% of cases occur in children, diagnosed mainly within the first 10 years, while adult-onset IPH often represents undiagnosed childhood cases as observed by Morgan et al. [2]. There is a slight male predominance in adult-onset IPH [3]. Familial clustering suggests potential genetic or environmental factors.

The etiology remains unknown, with theories including genetic, autoimmune, allergic, environmental, and metabolic factors as mentioned in multiple reports by Beckerman et al. [4], Thaell et al. [4], Iijima et al. [5], Lemley et al. [6], Wright et al. [7], Kayser et al. [8], and Fillet et al. [9].

During the acute phase, known as "IPH exacerbation," intra-alveolar bleeding leads to symptoms such as coughing, dyspnea, hemoptysis, and potentially respiratory failure. Hemoptysis is nearly universal during the course of the illness. Some patients may present with anemia, while others may have a normal physical examination. The chronic phase is marked by a gradual remission of symptoms, with features such as pallor, emaciation, hepatosplenomegaly, and occasionally a normal examination. Bilateral crackles and clubbing may be present, indicating lung fibrosis in the later stage. Both phases present repeatedly without any predominant sequence. On physical examination, anemia is the commonest one, with varying degrees of severity.

No single diagnostic test is confirmatory for IPH. Various diagnostic modalities are required during evaluation, such as imaging, sputum examination, BAL, TBLB, immunohistochemistry, and bone marrow biopsies. IPH lacks distinctive imaging features. Acute exacerbations display diffuse alveolar infiltrates on chest X-rays, predominantly in lower lung fields. High-resolution CT scans show comparable ground glass attenuation. Remission sees reabsorption, evolving into micronodular and interstitial reticular opacities with fibrosis as observed by Buschman et al. [10]. Technetium-99m (99mTc)- or chromium-51 (51Cr)-based perfusion scans reveal intra-alveolar bleeding, but their clinical utility is limited.

Without inflammatory syndromes, hepatic/renal issues, coagulopathies, or platelet abnormalities, a complete blood count indicates varying degrees of anemia. Diffuse alveolar hemorrhage (DAH) leads to pulmonary iron sequestration, causing sideropenic, microcytic anemia. Plasma ferritin levels may be normal or elevated as mentioned by Milman et al. [11]. Bone marrow biopsies reveal hyperplastic erythropoiesis and inadequate iron reserves as observed by Ali et al. [12]. Sputum examination, less sensitive, utilizes Prussian blue and hematoxylin and eosin stains for intra-alveolar hemorrhage. Inducing sputum in a non-responsive patient is risky. Bronchoalveolar lavage (BAL) is more diagnostically accurate than sputum tests as observed by Danel et al. [13]. Alveolar macrophages, containing intact erythrocytes, hemosiderin, and occasionally neutrophils, predominate in affected areas.

Immunofluorescence or immunohistochemistry is imperative for identifying immunoglobulins or immune complexes. Diagnostic testing encompasses anti-glomerular basement membrane (GBM) antibodies, anti-double-stranded DNA, antiphospholipid antibodies, and others. The macroscopic manifestation of "brown induration" in the lungs signifies iron infiltration and fibrosis. Chronic IPH is characterized by collagen deposition, hypertrophic type 2 pneumocytes, and thickened alveolar walls under microscopic examination. Pulmonary function tests show restrictive patterns as mentioned by Allue et al. [14]. The diffusing capacity of the lungs for carbon monoxide (DLCO) can be increased during acute phases and low or normal in chronic phases.

Treatment modalities include splenectomy and systemic glucocorticoids, with prednisolone recommended at 1 mg/kg/day. Chronic oral corticosteroids confer benefits by reducing fibrogenesis and exacerbations. Immunosuppressive drugs such as methotrexate, hydroxychloroquine, azathioprine, and cyclophosphamide are utilized, with azathioprine plus corticosteroids demonstrating efficacy as observed by Airaghi et al. [15].

In this patient, initially suspected of pulmonary tuberculosis, IPH was conclusively diagnosed through negative TB tests, bronchoalveolar lavage staining, and transbronchial lung biopsy. Iron deficiency anemia was diagnosed without urinary or gastrointestinal etiologies. The responsiveness to steroids and subsequent radiological resolution affirmed the diagnosis of IPH, underscoring the meticulous diagnostic steps essential for a definitive conclusion.

## Conclusions

IPH is a rare condition of unknown etiology, which primarily affects the young population. A combination of symptoms including cough, dyspnea, hemoptysis, alveolar infiltrates, and increasing anemia should raise suspicion of IPH after excluding the etiology of DAH. The diagnosis is based on the presence of hemosiderin-laden macrophages in the alveoli revealed by BAL and TBLB without signs of capillaritis/vasculitis, granulomatous inflammation, immunoglobulin deposition, or immunological complexes. Hence, the role of fiberoptic bronchoscopy and TBLB is crucial in the diagnosis of IPH.
